# Peroxisome Proliferator-Activated Receptor Delta (PPARδ) as a Metabolic Gatekeeper of Cerebrovascular Integrity

**DOI:** 10.1007/s12031-026-02576-z

**Published:** 2026-07-30

**Authors:** Shuhei Shiino, Crissey Pascale, Khashaiar Motazedian, Emma Lesser, Aaron Dumont, Alejandra N. Martinez

**Affiliations:** 1 Tulane University School of Medicine, 1430 Tulane Avenue, New Orleans, LA USA; 2 Department of Neurosurgery, Tulane Medical Center, 1430 Tulane Ave, New Orleans, LA USA; 3 Department of Neurosurgery, Tulane Center for Clinical Neurosciences, Tulane University School of Medicine, 1430 Tulane Ave, New Orleans, LA 70112 USA

**Keywords:** Mitochondrial function, Metabolism, Blood-brain barrier, Fatty acid oxidation, Vascular endothelial cells

## Abstract

The peroxisome proliferator-activated receptor delta (PPARδ) is a nuclear receptor highly expressed in vasculature and serves a central role in coordinating vascular and metabolic homeostasis across multiple physiological systems. Recent studies have highlighted PPARδ’s remarkable ability to regulate endothelial fatty acid oxidation, enhance metabolic flexibility, and modulate angiogenic responses critical for vascular adaptation and repair. Beyond its metabolic functions, PPARδ orchestrates multiple protective pathways that preserve cerebrovascular homeostasis, including endothelial repair, maintenance of blood-brain barrier integrity, mitochondrial biogenesis, and stability of the neurovascular unit, collectively enhancing endothelial resilience to stress. Dysregulation of PPARδ signaling has been implicated in various vascular and neurodegenerative disease states, underscoring its clinical relevance. In this literature review, we synthesize current findings on PPARδ-mediated signaling pathways and evaluate its potential as a promising therapeutic target. We further explore mechanistic roles across pathological contexts, as well as discuss emerging translational strategies aimed at harnessing PPARδ modulation for vascular and neuroprotective therapies.

**Graphical Abstract:**

**Figure:**
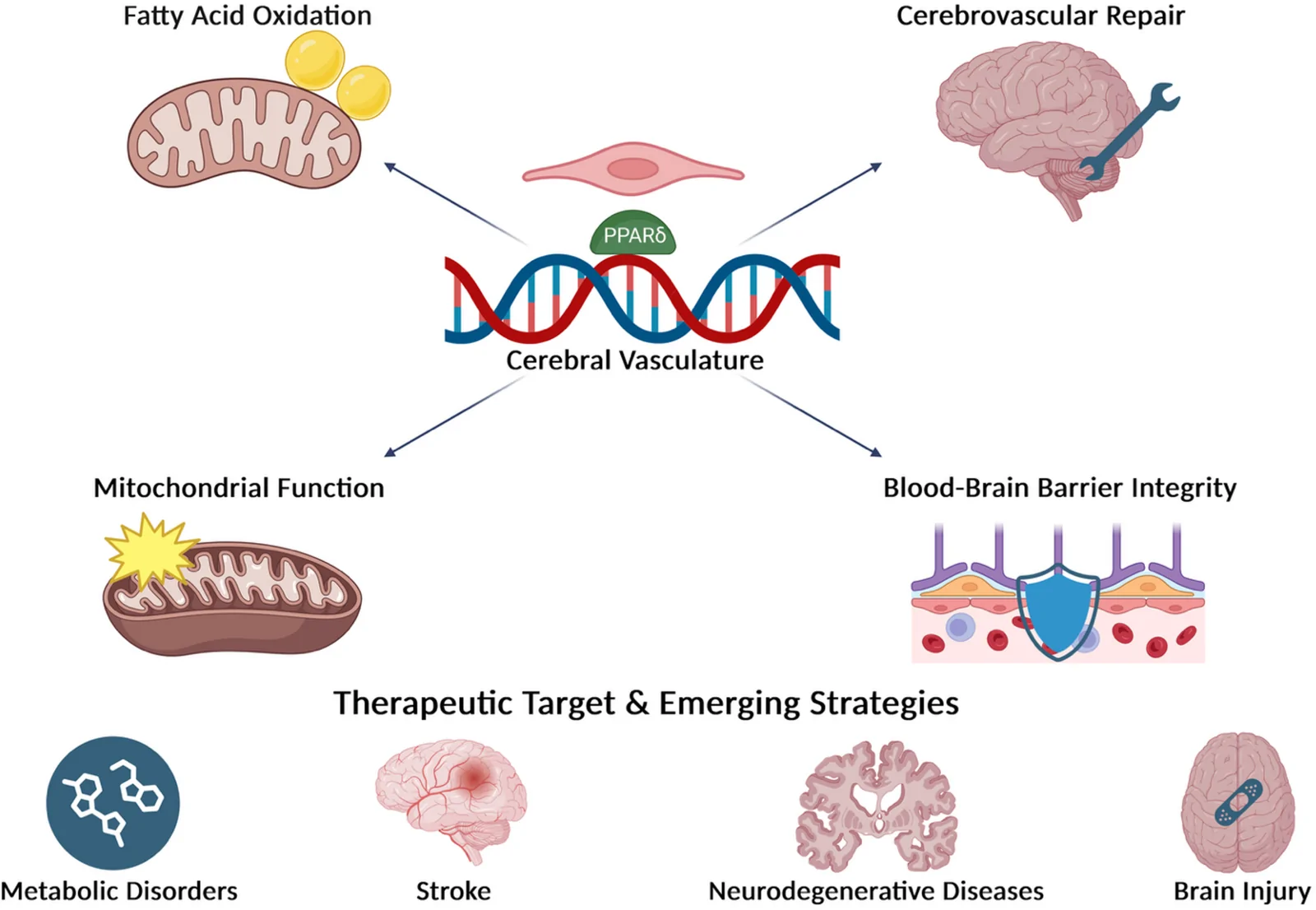

## Introduction

The peroxisome proliferator-activated receptors (PPAR) belong to a nuclear receptor superfamily comprising three main subtypes—PPARα, PPARβ/δ, and PPARγ (Wang 2008). Similar to its α and γ counterparts, PPARδ functions as a ligand-activated transcription factor that mediates diverse metabolic and cellular processes (Liu et al. 2018). Notably, the PPARδ isoform is highly expressed in the vasculature, where it senses lipid-derived ligands to assemble gene networks essential for the maintenance of endothelial homeostasis (Hwang et al. 2014). Upon activation by endogenous fatty acids or synthetic agonists, PPARδ forms a heterodimer with the retinoid X receptor (RXR) and binds peroxisome proliferator response elements (PPREs) to modulate gene transcription (Tugwood et al. 1992). PPARδ has been identified as a principal regulator of vascular homeostasis, acting through suppression of inflammatory pathways, enhancing metabolic efficiency, and attenuation of pathological leakage (Wawrzyniak et al. 2015). These findings suggest that PPARδ plays a role in cerebrovascular pathogenesis and highlight its promise as a therapeutic target for restoring neurovascular function in a variety of conditions involving ischemia and neurovascular inflammation (Titus et al. 2024; Wawrzyniak et al. 2015). Although previous studies have demonstrated the neuroprotective effects of PPARδ, this review reframes PPARδ as a key regulator of cerebrovascular function, underscoring its potential as a therapeutic target for neurovascular and metabolic brain disorders.

## PPARδ As a Driver of Endothelial Fatty Acid Oxidation (FAO)

PPARδ regulates endothelial cell (EC) function through multiple mechanisms that enhance resistance to cellular stress, including suppression of oxidative stress, inflammation, apoptosis, and thrombogenic signaling, while modulating vascular cell proliferation and survival (Ding et al. 2014). In the vascular endothelium, PPARδ exerts these effects in part through regulation of fatty acid oxidation (FAO), a metabolic pathway critical for endothelial energy homeostasis and angiogenic capacity (Liu et al. 2018). The metabolic role of PPARδ in ECs is highly context-dependent, varying with the physiological state of the endothelium. In a study utilizing a stable, non-proliferative monolayer of human umbilical vein endothelial cells (HUVECs), selective activation of PPARδ by an agonist suppressed both glycolysis and FAO. Conversely, during active tubulogenesis in vitro, PPARδ activation promoted FAO (Faulkner et al. 2020). These findings illustrate PPARδ’s ability to tailor endothelial metabolism to the functional demands of quiescent versus angiogenic states, rather than exerting a uniform metabolic effect. Moreover, although derived from peripheral endothelial models such as HUVECs, these observations provide a mechanistic framework that is broadly informative for endothelial biology and underscore the dynamic capacity of PPARδ to reprogram endothelial metabolism, directing FAO when additional energy is required for angiogenic processes.

PPARδ promotes FAO in ECs by upregulating enzymes central to mitochondrial energy metabolism (Toral et al. 2015a, b). In the mitochondrial transport of long-chain fatty acids during FAO, the rate-limiting step is controlled by the enzyme carnitine palmitoyltransferase-1 (CPT-1) (Toral et al. 2015b). In rodent models, fasting selectively activates PPARδ in brain endothelial cells (BECs), while pharmacologic stimulation with the agonist GW501516 produces a dose-dependent increase in *Cpt1a* transcription and upregulates additional FAO-associated enzymes, including acyl-CoA dehydrogenase long-chain (ACADL) and short-chain (ACADS) (Chasseigneaux et al. 2024). Growing evidence shows that PPARδ activation upregulates pyruvate dehydrogenase kinase 4 (PDK4), a key metabolic switch that inhibits pyruvate dehydrogenase, thereby reducing glucose oxidation and promoting reliance on FAO (Wu et al. 2022b; Zhang et al. 2017). In parallel, PPARδ induces uncoupling protein 2 (UCP2), which limits mitochondrial reactive oxygen species (ROS) production, improves mitochondrial efficiency, and contributes to endothelial protection (Toral et al. 2016).

To investigate the role of PPARδ in endothelial homeostasis, a recent study employed two complementary strategies in hindlimb ischemia (HLI) models. First, HLI was performed in EC-specific *Pparδ* knockout mice. Second, HLI was induced in obese mice fed a high fat diet that received EC-targeted Adeno-associated virus type 1 (AAV1) vector to overexpress PPARδ (Wu et al. 2022a). Single-cell RNA sequencing (scRNA-seq) analysis showed that *Pparδ* knockout hindered injury recovery by impairing angiogenesis, compromising endothelial integrity, and promoting inflammation, whereas endothelial PPARδ overexpression via AAV1 enhanced perfusion, increased capillary density, reduced inflammation, and improved muscle regeneration in both lean and obese ischemic mice (Wu et al. 2022a). Taken together, these findings position PPARδ as a critical metabolic regulator that dynamically aligns endothelial energy metabolism with functional demands through the promotion of FAO.

## PPARδ-Mediated Cerebrovascular Repair

Among the PPAR subtypes, PPARδ is uniquely characterized by its role in vascular remodeling and neurovascular responses during the repair phase following injury. For instance, PPARδ coordinates multiple protective mechanisms within the neurovascular unit (NVU) by enhancing endothelial progenitor cell (EPC) function to accelerate vascular repair (Piqueras et al. 2007), suppressing acute inflammatory mediators to reduce tissue damage and edema (Chehaibi et al. 2017), and promoting neural differentiation (Yu et al. 2012). Furthermore, selective activation of PPARδ safeguards blood-brain barrier (BBB) integrity by downregulating matrix metalloproteinase-9 (MMP-9) and upregulating critical tight junction proteins like claudin-5, occludin, and ZO-1 (Ouyang et al. 2026). Effectively orchestrating these complex regenerative processes, however, requires highly coordinated cellular and metabolic adaptations, particularly when overcoming cerebrovascular injury triggered by ischemia, intracerebral hemorrhage (ICH), aneurysm rupture, or chronic small-vessel diseases.

In ECs, FAO accounts for only about 5% of total ATP production under basal conditions (De Bock et al. 2013). However, as a restorative metabolic pathway, FAO supports essential EC functions including redox homeostasis (Kalucka et al. 2018), DNA synthesis (Harjes et al. 2016; Schoors et al. 2015), acetyl-CoA generation for epigenetic regulation (Simeroth and Yu 2024), and ATP generation (Patella et al. 2015). While these functions represent core survival mechanisms common to all vasculature, metabolic flexibility becomes especially critical in BECs, which must rapidly restore barrier integrity and sustain neurovascular homeostasis following an insult.

This heightened metabolic demand reflects the specialized phenotype of BECs. Although many foundational studies of endothelial FAO were performed in peripheral models, BECs have evolved unique structural and metabolic adaptations required to support the blood–brain barrier (BBB) (Daneman and Prat 2015). In addition to their abundant expression of tight junction proteins and exceptionally low rates of transcytosis, BECs display a distinct metabolic programming (Dyatlova et al. 2022). Specifically, they contain a significantly higher density of mitochondria and depend more heavily on oxidative phosphorylation (OXPHOS) than their peripheral counterparts (Banks and Rhea 2021). This enhanced mitochondrial reliance sustains the substantial energetic demands associated with preserving tight junction integrity, driving active transport, and preserving redox balance essential for BBB function (Eelen et al. 2015). Consequently, post-injury cerebrovascular recovery depends on this unique mitochondrial machinery to drive the metabolic flexibility needed to restore barrier integrity, perfusion, and neurovascular homeostasis. By fueling the TCA cycle, supporting endothelial proliferation, and limiting oxidative stress, FAO has gained increasing attention as a potential therapeutic target for promoting cerebrovascular repair and neurovascular recovery (Draoui et al. 2017; Liu et al. 2018).

## PPARδ in Blood-Brain Barrier (BBB)

Disruption of the BBB contributes to the progression of Alzheimer’s disease, multiple sclerosis, aneurysms, and ischemic stroke by permitting inflammatory mediators and immune cells into the central nervous system (CNS) (Alkhalifa et al. 2023; Llull et al. 2025; Shimizu and Nakamori 2024; Zierfuss et al. 2024). BBB breakdown is increasingly regarded as a hallmark of acute neuroinflammatory responses in these disorders, exacerbating neuronal injury and accelerating disease progression. Across multiple rodent models of CNS injury, PPARδ has emerged as a pleiotropic regulator of neurovascular integrity and inflammatory signaling. Pharmacological activation of PPARδ with agonists such as GW0742 and GW501516 suppressed tumor necrosis factor-alpha (TNF-α)-induced expression of endothelial adhesion molecules, including VCAM-1 and E-selectin, which are critical mediators of leukocyte recruitment and BBB destabilization (Fan et al. 2008). In a mouse model of experimental autoimmune encephalomyelitis, treatment with GW0742 reduced clinical severity and demyelination via suppression of inflammatory cell infiltration and preservation of BBB integrity (Polak et al. 2005).

Similarly, in a focal cerebral ischemia model, GW0742 significantly upregulated the expression of key tight junction proteins, such as claudin-5, occludin, and ZO-1, resulting in reduced vascular permeability within ischemic infarct regions (Chehaibi et al. 2017). Furthermore, adenovirus-guided overexpression of PPARδ in a rat endovascular perforation subarachnoid hemorrhage model led to significant improvement in neurological deficits, brain edema, BBB impairment, and neural cell apoptosis (Teng et al. 2016). Beyond its anti-inflammatory and barrier-stabilizing effects, PPARδ also regulates metabolic adaptations at the BBB, as evidenced by its upregulation of the ketone body transporter, monocarboxylate transporter 1 (MCT1), in rat brain endothelial cells during fasting, suggesting a role in coordinating metabolic stress responses at the neurovascular interface (Chasseigneaux et al. 2024).

PPARδ activation also exerts its protective effect through the inhibition of MMP-9, a class of zinc-dependent endoproteases that weakens the BBB by degrading structural proteins in the extracellular matrix (Tang et al. 2020; Yin et al. 2011a). Reduction in MMP-9 activity and expression effectively lowers brain edema and BBB leakage in intracranial hemorrhage mouse models (Wu et al. 2010). Nuclear factor kappa B (NF-κB), a transcriptional factor that increases MMP-9 expression and overall inflammation, has its signals disrupted by PPARδ activation, resulting in reduced brain edema (Di Paola et al. 2010). Moreover, activation of PPARδ has been shown to have anti-apoptotic effects in ECs and attenuate alterations in tight junction protein expression, which leads to leakage, revealing PPARδ’s ability to sustain BBB function under pathological conditions (Iwashita et al. 2007; Luissint et al. 2012; Wu et al. 2010). Another study conducted by Mondal et al. discovered that oral administration of gemfibrozil, a known ligand of another isoform of PPAR receptors, PPARα, was ineffective in maintaining BBB integrity in PPARδ knockout rodent models, further highlighting the direct linkage of PPARδ activation to the preservation of BBB function (Mondal et al. 2024). Thus, mechanisms that protect the BBB through direct upregulation of PPARδ activation and expression may be promising targets in developing novel therapies for ischemic stroke and other neurological diseases.

## PPARδ in the Neurovascular Unit (NVU)

The NVU is dynamic structural complex composed of neurons, glial cells, brain microvascular endothelial cells (BMECs), pericytes, and the extracellular matrix. Together, these cellular and structural components control the permeability of the BBB and maintain the tightly controlled microenvironment required for normal neuronal function, thereby protecting the brain from blood-borne, endogenous, and exogenous insults (Gong et al. 2025). Under pathological conditions such as stroke, NVU homeostasis becomes severely disrupted, leading to BBB breakdown and irreversible neuronal excitotoxicity, injury, and death (Yang et al. 2025).

Previous studies show PPARδ is broadly expressed throughout both the parenchymal and vascular compartments of the brain, where it serves as an important regulator of intercellular communication within the NVU (Hall et al. 2008; Woods et al. 2003; Yang et al. 2025). In astrocytes, activation of PPARδ has a protective effect on oxidative injury by enhancing degradation of the transcription factor ATMIN and reducing ROS production for the restoration of peroxisomal homeostasis (Yang et al. 2025). In rodent models of focal cerebral ischemia, PPARδ-null mice exhibit significantly larger infarct volumes compared with wild-type controls, further highlighting the essential role of PPARδ in preserving NVU integrity and resilience following cerebral injury (Arsenijevic et al. 2006; Pialat et al. 2007).

PPARδ also plays a central role in regulating the vascular and immune components of the NVU. Unlike PPARα and PPARγ, which primarily exert anti-angiogenic effects, PPARδ uniquely promotes angiogenesis by enhancing VEGFR expression and activating pro-angiogenic signaling pathways that support endothelial survival, post-stroke revascularization, and vascular repair (Jiang et al. 2019; Strosznajder et al. 2021; Yin et al. 2011b). At the same time, PPARδ activation attenuates neuroinflammation by suppressing microglial polarization toward the pro-inflammatory state, thereby limiting secondary NVU disruption and tissue injury under pathological stress (Barish et al. 2008; Bishop-Bailey and Bystrom 2009). Collectively, these findings position PPARδ as a key regulator of NVU integrity and cellular crosstalk, integrating vascular repair, glial protection, immune modulation, neuronal survival, and vascular resilience to preserve neurovascular homeostasis following brain injury (Table 1).

**Table 1:** Key studies associated with PPARδ activation

Category	Key Outcomes	Mechanism
Fatty Acid Oxidation (FAO) (Chasseigneaux et al. 2024; Ding et al. 2014; Faulkner et al. 2020; Toral et al. 2015a, b, 2016; Wu et al. 2022a, b; Zhang et al. 2017)	Facilitates angiogenesis, augments endothelial adaptability, and optimizes tissue perfusion in a context-dependent manner	Upregulates CPT-1, ACADL, ACADS, PDK4, and UCP2
Cerebrovascular Repair (Banks and Rhea 2021; Daneman and Prat 2015; De Bock et al. 2013; Draoui et al. 2017; Dyatlova et al. 2022; Eelen et al. 2015; Harjes et al. 2016; Kalucka et al. 2018; Patella et al. 2015; Schoors et al. 2015; Simeroth and Yu 2024)	Sustains endothelial survival, enforces barrier integrity, and builds resistance to oxidative stress after injury	Elevates acetyl-CoA to fuel TCA cycle and OXPHOS, fuels nucleotide synthesis, and preserves redox homeostasis
Blood-brain barrier (BBB) (Abbott et al. 2010; Chehaibi et al. 2017; Di Paola et al. 2010; Fan et al. 2008; Iwashita et al. 2007; Luissint et al. 2012; Mondal et al. 2024; Polak et al. 2005; Tang et al. 2020; Teng et al. 2016; Wu et al. 2010; Yin et al. 2011a)	Preserves BBB integrity, and attenuates neuroinflammation and cerebral edema	Suppresses adhesion molecules and NF-κB–MMP-9 signaling, strengthens tight junction proteins, and supports metabolic adaptation
Neurovascular Unit (NVU) (Arsenijevic et al. 2006; Barish et al. 2008; Bishop-Bailey and Bystrom 2009; Gong et al. 2025; Hall et al. 2008; Jiang et al. 2019; Pialat et al. 2007; Strosznajder et al. 2021; Woods et al. 2003; Yang et al. 2025; Yin et al. 2011b)	Maintains neurovascular homeostasis, enhances post-stroke revascularization, and supports neuronal survival after injury	Enhances VEGFR-mediated pro-angiogenic signaling, suppresses pro-inflammatory microglial polarization, and coordinates vascular-glial-immune crosstalk within the NVU

## PPARδ as a Therapeutic Target

The mechanistic relevance of PPARδ is further strengthened by its endogenous ligand biology. PPARδ can be activated by a diverse range of fatty acids and lipid-derived mediators, including palmitic, stearic, and oleic acids at physiologically relevant concentrations (Honda et al. 2025; Li et al. 2008), as well as electrophilic nitrated lipids such as nitro-oleic acid and nitro-linoleic acid (Ferreira et al. 2012). Together, these findings support the concept that PPARδ is not only pharmacologically targetable, but also endogenously engaged within lipid-rich and oxidative stress–associated vascular microenvironments relevant to cerebrovascular disease.

Despite a strong mechanistic rationale, translational evidence supporting PPARδ-targeted therapies in cerebrovascular disease remains limited, as studies largely focus on systemic metabolic endpoints rather than neurovascular outcomes. PPARδ agonists were initially developed for metabolic indications, including dyslipidemia, insulin resistance, and mitochondrial myopathies (Fedorova et al. 2013; Feng et al. 2014; Matsushita et al. 2011; Niu et al. 2015; Saibil et al. 2019; Tanaka et al. 2003; Wagner and Wagner 2022). Although several selective investigational agonists demonstrated early preclinical promise, their clinical translation has been hindered by efficacy or safety limitations. For instance, Mavodelpar (REN001) and Bocidelpar (ASP0367) failed to meet efficacy endpoints in clinical trials (Authors 2023, Iwai et al. 2025) while the clinical development of GW501516 was terminated due to toxicity and oncogenic risk (Wagner and Wagner 2020). Furthermore, while preclinical evaluation of GW0742 indicates neuroprotective potential in models of hippocampal toxicity (An et al. 2016) and epilepsy (Zubareva et al. 2024), these investigations have not yet extended to cerebrovascular pathologies.

Seladelpar (MBX-8025) is the only FDA-approved selective PPARδ agonist, having gained approval in August 2024 for primary biliary cholangitis (PBC) (Hoy 2024). However, definitive clinical evidence demonstrating cerebrovascular protection or vascular repair in human ischemic stroke, ICH, or small vessel disease is currently lacking. This disconnect highlights a profound translational gap, underscoring an urgent need for an integrated, cerebrovascular-focused framework that links ligand biology, mitochondrial metabolism, and endothelial signaling directly to therapeutic development (Table 2).

**Table 2:** PPARδ agonists in past and current clinical trials

Ligand/Agonist	Type/Source	Potency (EC_50_)	Indication/Study	Development Status
Mavodelpar (REN001)	Synthetic selective PPARδ agonist	~ 31nM	Genetic mitochondrial myopathies(Fedorova et al. 2013)	Phase 2b discontinued in 2023 NCT04535609(December 14, 2023)
Bocidelpar (ASP0367; MA-0211)	Synthetic PPARδ modulator	~ 7.8nM	Primary mitochondrial myopathies(Feng et al. 2014) Duchenne muscular dystrophy(Bell et al. 2019)	Phase 2 discontinued in 2024 NCT04641962(Iwai et al. 2025)
GW501516	Synthetic highly selective PPARδ agonist	~ 1nM	Metabolic syndrome(Wagner and Wagner 2022)^,^ (Saibil et al. 2019)^,^ (Tanaka et al. 2003) Acute liver failure(Lim and Kwak 2024) Running endurance(Chen et al. 2015) Pulmonary hypertension(Liu et al. 2013)	Preclinical terminated due to increased cancer risk(Wagner and Wagner 2020) Banned as performance enhancer
GW0742	Synthetic highly selective PPARδ agonist	~ 1nM	Antidiabetic(Niu et al. 2015) Diabetic nephropathy(Matsushita et al. 2011) Cardiac hypertrophy(Cheng et al. 2018) Aβ-42-induced hippocampal neurotoxicity(An et al. 2016) Temporal lobe epilepsy(Zubareva et al. 2024)	Preclinical only; Banned as performance enhancer
Seladelpar (MBX-8025)	Selective PPARδ.agonist	~ 2nM	Primary biliary cholangitis(Hoy 2024)	FDA approved in August 2024

## Conclusion

In conclusion, activation of PPARδ plays an essential role in guiding endothelial metabolism, coordinating cerebrovascular repair, enhancing mitochondrial function, and maintaining BBB integrity. Preclinical studies provide compelling evidence supporting its therapeutic potential across multiple disease contexts, and ongoing research on both endogenous and synthetic PPARδ agonists holds promise for overcoming current translational challenges. Continued research in PPARδ modulation, along with optimizing dosage and delivery, may unveil novel strategies to combat metabolic, cardiovascular, and cerebrovascular disorders.

## Data Availability

No datasets were generated or analysed during the current study.
